# HScore as a diagnostic tool in ERBB2 equivocal (immunohistochemistry 2+) breast cancer

**DOI:** 10.1093/labmed/lmag006

**Published:** 2026-04-08

**Authors:** Tasnem Alsebai, Muhammed Yaman Swied, Ricardo Cossyleon, Kathy Robinson, Kristin Delfino, John Gao, Krishna Rao

**Affiliations:** Department of Internal Medicine, Southern Illinois University School of Medicine, Springfield, IL, United States; Department of Internal Medicine, Southern Illinois University School of Medicine, Springfield, IL, United States; Department of Internal Medicine at Simmons Cancer Institute, Center for Clinical Research, Southern Illinois University School of Medicine, Springfield, IL, United States; Department of Internal Medicine at Simmons Cancer Institute, Center for Clinical Research, Southern Illinois University School of Medicine, Springfield, IL, United States; Department of Surgery, Center for Clinical Research, Southern Illinois University School of Medicine, Springfield, IL, United States; Department of Laboratory Medicine, Springfield Memorial Hospital, Springfield, IL, United States; Department of Internal Medicine at Simmons Cancer Institute, Southern Illinois University School of Medicine, Springfield, IL, United States

**Keywords:** ERBB2, IHC, breast cancer

## Abstract

**Introduction:**

*ERBB2* is amplified or overexpressed in 15% to 20% of primary invasive breast cancers. Despite the aggressive nature of ERBB2-positive breast cancers, the development of anti-ERBB2 therapy has resulted in better prognoses. HScore is a scoring system that broadens immunohistochemistry (IHC) results into a quantitative range. The aim of this retrospective study was to evaluate whether the HScore can be used to predict all ERBB2 IHC-equivocal cases as positive or negative without the need for fluorescence in situ hybridization confirmation.

**Methods:**

Image acquisition and processing tools were used on previously collected tissue slides from patients with ERBB2 IHC 2+ breast cancer from 2014 to 2023. After image acquisition, HScore values were calculated using variables from image analysis software.

**Results:**

The mean HScore values in ERBB2-positive cases were higher (3.06) than in ERBB2-negative cases (2.62). Receiver operating characteristic curve analysis yielded an area under the curve of 0.668 (95% CI, 0.523-0.812), indicating poor predictive accuracy. The optimal HScore cutoff of 2.6007 provided moderate sensitivity (0.77) but limited specificity (0.58).

**Discussion:**

Although the HScore showed moderate predictive accuracy, it lacked the reliability to replace fluorescence in situ hybridization as the gold standard. Future research should focus on refining the methodology and integrating it with other diagnostic approaches to enhance accuracy and clinical utility.

## Introduction

Breast cancer is the most common cancer in women and the second-leading cause of cancer-related deaths in women worldwide.^1^ Although breast cancer is commonly associated with women, it can also occur in men, with approximately 2800 cases diagnosed annually in the United States.^2^ Breast cancer–associated biomarker identification, including estrogen receptor, progesterone receptor, and ERBB2, dramatically improved the ability to predict patient prognosis and tailor treatments. The ERBB2 protein is a receptor tyrosine kinase located on chromosome 17q21 that participates in controlling how breast cells divide and repair themselves.^3^  *ERBB2* gene amplification or ERBB2 protein overexpression, however, which is present in 15% to 20% of primary invasive breast cancers, can lead to breast cells to proliferate at an increased rate.^3^ In addition, overexpression of ERBB2 is clinically relevant due to its aggressive nature and historically poor prognosis. Fortunately, the development and use of anti-ERBB2 therapy has dramatically improved the prognosis for ERBB2-positive breast cancer, making early and accurate ERBB2 testing critical for effective disease management.

Immunohistochemistry (IHC) and fluorescence in situ hybridization (FISH) are the 2 primary methods for assessing ERBB2 status in breast cancers, with chromogenic in situ hybridization, including silver in situ hybridization, as alternative methods for determination of ERBB2 status. Immunohistochemistry determines the ERBB2 receptor expression status at the cancer cell surface, while FISH detects *ERBB2* gene amplification within the cell nucleus.^4^ Currently, FISH is considered the gold standard for determining ERBB2 status because of its high sensitivity and specificity; however, it is more expensive and takes longer to complete than IHC, so it is often reserved for confirmation after an equivocal IHC result. For this reason, IHC is generally preferred as the initial test because of its quicker turnaround time and practical advantages.^5^

According to the 2023 American Society of Clinical Oncology/College of American Pathologists evaluation criteria, ERBB2 IHC results are interpreted based on both the intensity of staining and the percentage of stained tumor cells.^6^ Scores of 0 or 1+ indicate no staining or faint, incomplete staining and are considered ERBB2 negative. A score of 3+ means intense, complete staining on more than 10% of tumor cells and is classified as positive. Results with a score of 2+ are less clear: They show weak to moderate staining in more than 10% of cells or intense staining in fewer cells. These borderline cases, which make up about 5.8% of invasive breast cancers, usually require follow-up testing with FISH to determine ERBB2 status definitively.^7^ Unfortunately, FISH can slow down diagnosis, delay treatment decisions, and increase costs.^5^

The HScore is a metric that provides a means to extend the traditional IHC result into a broader quantitative range that accounts both for staining intensity and for percentage of positive cells.^8^ The HScore can be calculated using ImageJ, open-source image analysis software used in biological and medical research to analyze and interpret imaging data. ImageJ quantifies the staining intensity by analyzing pixel values within the area of interest. Unlike manual assessment by pathologists, which is likely to be more subjective and may vary between observers, the HScore provides a more objective and reproducible measurement. If proven effective, the HScore could substantially reduce the need for FISH confirmation in equivocal cases, speeding up diagnosis and reducing costs. This retrospective study aims to explore whether the HScore can achieve this goal by accurately predicting ERBB2 status in IHC 2+ equivocal breast cancer cases.

## Methods

Our single-center retrospective study enrolled patients older than 18 years of age who were diagnosed with breast cancer and had ERBB2 IHC 2+ status confirmed either positive or negative by FISH from January 2014 to December 2023. The clinicopathologic variables investigated included demographic characteristics (age, sex, race, ethnicity) and pathologic characteristics (breast cancer type, ERBB2 status, estrogen receptor and progesterone receptor status, pathologic TNM stage, and Nottingham score and grade). All data were collected through chart review from electronic health records. In addition, we reviewed the biopsy pathology slides that were used for the diagnosis of the ERBB2 IHC 2+ breast cancer.

To reduce interobserver variability, all image capture and ImageJ-based HScore calculations were performed by a single trained reviewer blinded to the final ERBB2 FISH results. Imaging parameters, including magnification, illumination, white balance, and resolution, were standardized before image capture. Thresholds in ImageJ were applied using a consistent, predefined method based on the mean of the minimum and maximum pixel intensities within the stained regions. This standardized workflow ensured measurement consistency and enhanced comparability among specimens.

To determine the HScore values of the stained IHC specimens, we first captured images of the pathology slides through Brightfield images at ×10 magnification using an Olympus Microscope BH-2 device. Images were captured using a Motic Moticam 1000 1.3-megapixel camera attached to the microscope and connected to a computer. All the images were acquired using Motic Images Plus, version 3.1, software and saved in JPEG format. Before capturing the images, the color density, background, white balance, and resolution were standardized. The images were captured from 3 different fields per slide and within the region of the tumor. When we had obtained the pictures, we used a computer-aided image analysis program developed by the National Institutes of Health, ImageJ, which can be used for IHC analysis. With this software, the area of interest was selected by a threshold, which was the mean of the minimum and maximum values of the pixels related to the IHC-stained area, in such way that only the areas of interested were included in the analysis.

During image processing, we obtained 2 key numeric values from the tissue slide: the mean intensity of staining and the percentage of the tissue area stained. We then calculated the HScore using the following formula:


HScore=∑ pi (I+1)

where Σ pi is expressed as optical density (log [255/mean intensity]) and *I* is the percentage area of staining. Of note, a mean intensity value of 0 depicts a more intense staining, which results in darker pixels (pixel value of 0 = black; pixel value of 255 = white).

To assess internal validity and the extent to which the HScore differentiates ERBB2 expression levels, additional analyses were performed on representative ERBB2 IHC 0, 1+, and 3+ cases. Ten cases from each category were analyzed using the same ImageJ workflow, with the same image acquisition settings, thresholding, and HScore calculations as used for the IHC 2+ group. Patients from the ERBB2 IHC 0, 1+, and 3+ groups were not included in the main analysis but were evaluated for the purpose of assessing HScore performance across different ERBB2 expression levels.

### Statistical analysis

Binary linear regression was used to assess the predictive value of continuous-variable HScore on the binary outcome of final FISH ERBB2 status. Odds ratios with 95% CIs were reported. Multiple-variable models were used to further adjust for possible confounders in the previously described model. To explore the prognostic relationship between the continuous HScore and final ERBB2 status, a cut point model was used. A graphical representation of the data was created to view the relationship between the variables. To determine whether a threshold effect exists and estimate an optimal cut point, a series of 2-sample *t* tests was conducted for multiple possible dichotomizations of the HScore value. Bonferroni correction was applied to each *P* value to correct for multiple testing. *P* < .05 was considered statistically significant.

## Results

A retrospective study was conducted to evaluate the demographic and pathologic characteristics of patients diagnosed with ERBB2 IHC+ 2 breast cancer between January 2014 and December 2023. A total of 331 patients with ERBB2 IHC 2+ breast cancer were initially identified. After excluding 149 patients because of missing clinical data or unavailable original IHC slides, 182 patients remained eligible for analysis. Based on confirmatory FISH testing, 169 cases were classified as ERBB2 negative and 13 cases as ERBB2 positive. Unless otherwise specified, analyses were performed on the entire cohort, with subgroup analyses stratified according to final ERBB2 status.

Among the eligible population with ERBB2 IHC 2+ breast cancer, 97.8% of patients were female and 2.2% were male, with a median age at diagnosis of 61 years (range, 23-93 years; SD, 13.26 years). Moreover, 85.7% of the patients identified as White, 4.4% as African American, 1.6% as other, and 8.2% declined to report this information, while 92.3% reported a non-Hispanic ancestry, 0.5% reported a Hispanic ancestry, and 7.1% declined to report this information.

**Figure 1:**
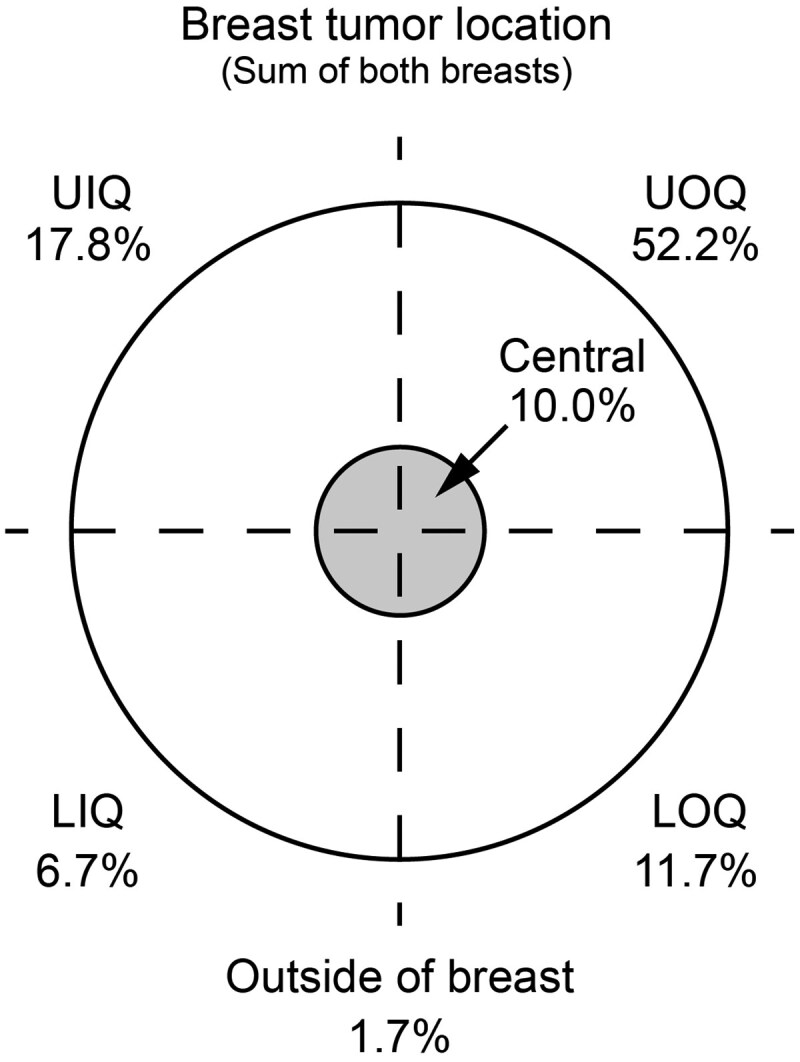
Breast tumor location. Figure depicts the incidence percentage, by quadrant location, of the primary breast tumor (sum incidence of both breasts). LIQ indicates lower inner quadrant; LOQ, lower outer quadrant; UIQ, upper inner quadrant; UOQ, upper outer quadrant.

At diagnosis, 83.5% of the patients underwent a core needle biopsy, while 15.9% had an excisional biopsy of the lesion and 0.5% had a fine-needle biopsy. Moreover, the primary lesion was located in the left breast in 56.0% of the patients; tumor location by quadrant can be found in Figure 1. Clinical staging revealed the following distribution: 57.7% stage I, 32.4% stage II, 8.2% stage III, and 1.6% stage IV. Detailed TNM staging can be found in Table 1. Pathologic analysis indicated that 84.1% of tumors were ductal carcinomas, 11.0% were lobular carcinomas, 0.5% were mixed ductal/lobular carcinomas, and 4.4% were other breast cancer subtypes. Comprehensive pathologic characteristics are summarized in Table 2.

**Table 1:** TNM staging (*n* = 182).

	X, No. (%)	0, No. (%)	1, No. (%)	2, No. (%)	3, No. (%)	4, No. (%)
T	0 (0.0)	2 (1.0)	108 (59.3)	58 (31.9)	10 (5.5)	4 (2.2)
N	10 (5.5)	133 (73.1)	27 (14.8)	9 (4.9)	3 (1.6)	—
M	9 (4.9)	170 (93.4)	3 (1.6)	—	—	—

**Table 2:** Pathologic characteristics.

		No.	%
Receptor status	Estrogen receptor positive	162	89.0
	Estrogen receptor negative	20	11.0
	Progesterone receptor positive	155	85.2
	Progesterone receptor negative	27	14.8
	ERBB2 positive (by FISH)	13	7.1
	ERBB2 negative (by FISH)	169	92.9
Nottingham score	3	0	0.0
	4	19	10.4
	5	20	11.0
	6	68	37.4
	7	29	15.9
	8	29	15.9
	9	14	7.7
	Not available	3	1.6

Abbreviation: FISH, fluorescence in situ hybridization.

**Figure 2:**
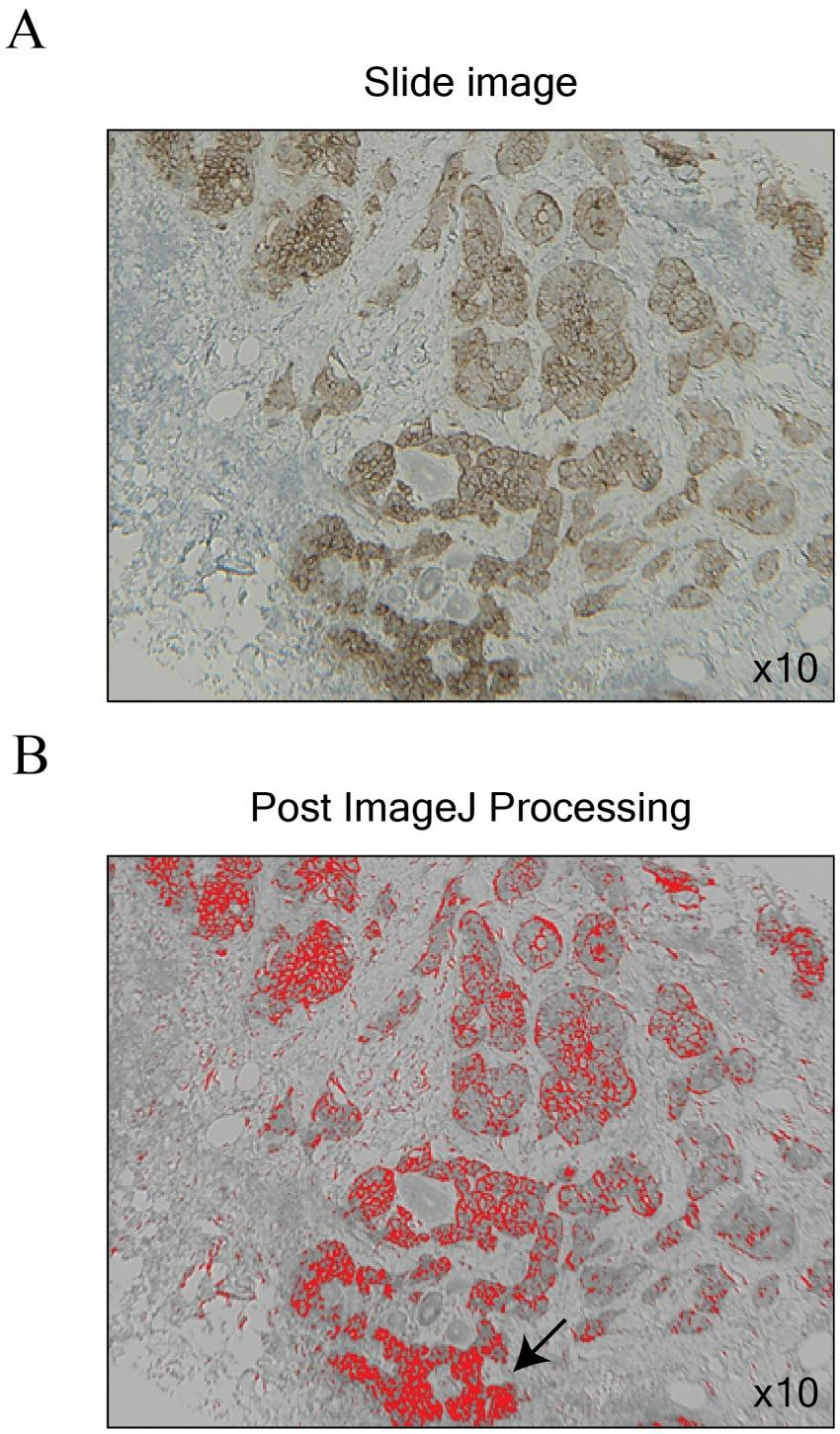
Processing of tissue slide images. **(A)** Sample image of a stained immunohistochemistry slide used to confirm ERBB2 status as 2+ (equivocal; ×10). **(B)** Same image as Figure 2A after ImageJ software processing to confirm proper selection of stained areas (red; ×10).

Tissue slides were photographed and processed as mentioned in the “Methods” section by a person unaware of the ERBB2 status of each patient. Image processing examples can be found in Figure 2. The mean intensity of the slides from the 169 patients with ERBB2-negative slides was 113.3 (range, 66.4-142.8; SD, 13.9) while the ERBB2-positive slides was 103.82 (range, 59.1-126.4; SD, 20.8). Moreover, the average percentage area of staining of ERBB2 negative slides was 6.3% (range, 2.6%-13.8%; SD, 1.9%) and 6.8% (range, 3.6%-11.9%; SD, 2.1%) in ERBB2-positive slides.

**Figure 3:**
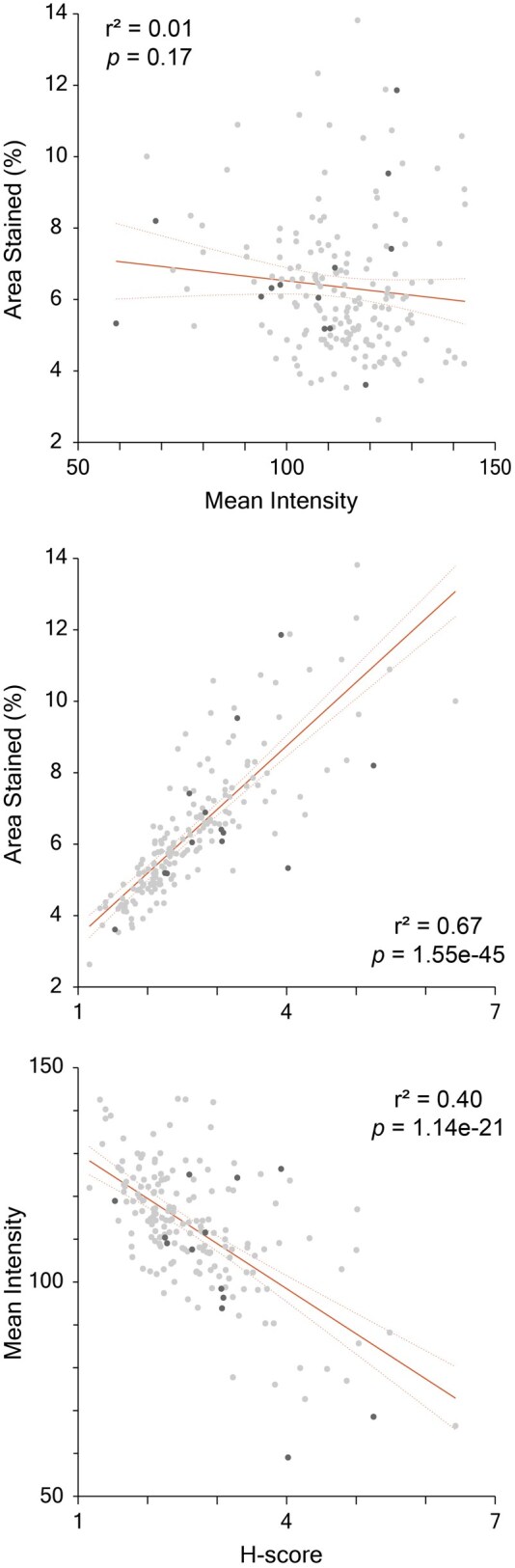
Correlation between percentage of area stained, intensity, and HScore. Light gray dots represent cases with proven ERBB2-positive status by fluorescence in situ hybridization. Black dots represent ERBB2-negative cases. Percentage of cases shown in parentheses.

The predictive utility of the HScore for ERBB2 status classification was assessed. The overall mean HScore for the entire population was 2.65, with ERBB2-positive cases exhibiting a mean HScore of 3.06 and ERBB2-negative cases a mean HScore of 2.62. By observing a correlation between the percentage of area stained with the resulting HScore as well as the mean intensity and HScore confirms that these parameters truly influenced the final HScore. Meanwhile, there was no correlation between intensity and percentage of area stained (Figure 3).

**Figure 4:**
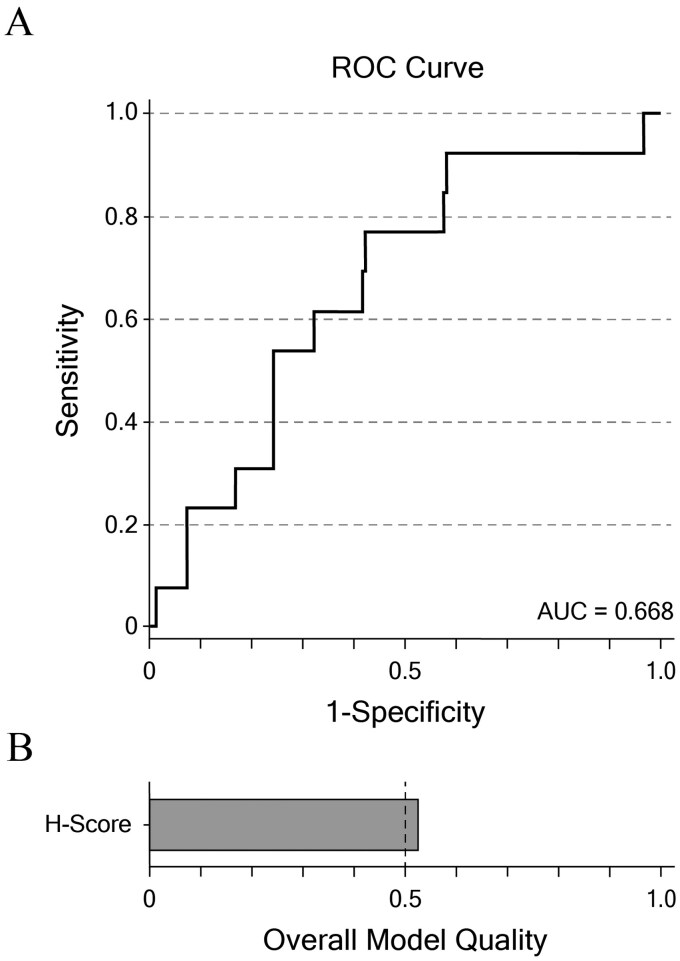
Model evaluation. **(A)** Receiver operating characteristic curve depicting the discriminatory power of the HScore. **(B)** Overall quality of the model to predict ERBB2 status using the HScore. AUC indicates area under the curve; ROC, receiver operating characteristic.

Receiver operating characteristic (ROC) curve analysis was performed to evaluate the discriminatory performance of the HScore. The area under the curve (AUC) was 0.668 (95% CI, 0.523-0.812; *P* = .023) (Figure 4), indicating moderate predictive ability. Although the result was statistically significant, the moderate AUC suggests that the HScore has limited accuracy as a stand-alone classifier for ERBB2 status. Using the Youden index, the optimal HScore cutoff was determined to be 2.6007, which corresponded to a sensitivity of 0.77 and a specificity of 0.58. The small number of ERBB2-positive cases in the cohort, however, may have constrained the overall predictive performance of the HScore. These findings indicate that although the HScore provides some discriminatory value, its utility as an independent predictor of ERBB2 status remains limited.

As part of the internal control analysis, HScore values were calculated for ERBB2 IHC 0, 1+, and 3+ cases (10 cases per group) using the same image analysis method applied to the IHC 2+ cohort. Mean HScore values generally increased with higher ERBB2 expression, with the highest values observed in IHC 3+ cases (Table 3).

**Table 3:** HScore comparison.

ERBB2 IHC	No.	HScore, mean	SD	Range
0	10	3.35	0.74	2.10-4.51
1+	10	2.56	0.97	1.51-4.55
2+	182	2.65	0.88	1.16-6.43
3+	10	4.02	1.43	2.46-6.75

Abbreviation: IHC, immunohistochemistry.

Our ROC analyses similarly showed good discrimination between IHC 3+ and IHC 2+ and 1+ cases but weaker performance among lower expression comparisons. Although pairwise comparisons establish statistically significant differences in average HScore values across ERBB2 IHC categories, ROC/AUC analyses provide complementary information by evaluating the discriminatory performance of the HScore at the individual case level. Our ROC analyses confirmed strong discrimination for IHC 3+ vs IHC 2+ and 1+ cases (AUC = 0.83) but demonstrated limited separation among IHC 0, 1+, and 2+ cases, highlighting substantial overlap within the ERBB2 low range that is not fully captured by group-level statistics alone (Table 4).

**Table 4:** ROC association statistics.

Group	AUC	SE	95% CI
1 vs 0	0.740	0.126	0.493-0.987
2 vs 0	0.760	0.076	0.612-0.908
3 vs 0	0.610	0.139	0.338-0.882
2 vs 1	0.549	0.107	0.338-0.759
3 vs 1	0.830	0.100	0.634-1.000
3 vs 2	0.832	0.054	0.727-0.938

Abbreviations: AUC, area under the curve; ROC, receiver operating characteristic.

Notably, the relatively higher mean HScore observed in IHC 0 cases may reflect technical challenges related to the narrow intensity range in this group, which can affect accurate differentiation between true staining and background signal.

## Discussion

Immunohistochemistry is a staining technique used to detect the ERBB2 protein in cancer cells by targeting specific antigens within tissue samples. The process begins by preparing the tissue to expose these antigen sites, followed by the application of monoclonal or polyclonal antibodies that bind to the ERBB2 protein. A chemical reaction then produces a color change, indicating ERBB2 receptors.^8^ Fluorescence in situ hybridization is a cytogenetic technique used to detect *ERBB2* gene amplification in breast cancer cells. This technique uses fluorescent DNA probes that bind to the *ERBB2* gene within the tumor cell nucleus. Increased fluorescence signals indicate gene amplification, which correlates with an aggressive tumor and is critical for determining treatment options.^8^ Because IHC is easy to perform and cost-effective, it is the primary method for determining ERBB2 status; however, variability in sensitivity and specificity has been reported among commercially available antibodies. Therefore, FISH serves as an alternative modality, offering higher sensitivity and specificity, particularly in cases where IHC results are equivocal.^9^

Studies comparing IHC and FISH have reported varying degrees of concordance between the 2 techniques. For instance, 1 study reported a concordance rate of 93.8% between IHC and FISH when assessing ERBB2 status, indicating moderate agreement.^10^ Another study highlighted that discordance rates were higher in cases with IHC 2+ results, suggesting that FISH may be more reliable in such equivocal cases.^11^ Ensuring accurate laboratory assessment of ERBB2 status is crucial because it supports appropriate treatment selection for breast cancer while minimizing the risk of adverse effects in women without ERBB2 amplification or overexpression.^12^

Although ERBB2 status can be directly tested by FISH, many laboratories favor the use of IHC as an initial approach, reserving FISH for confirmation in ERBB2 IHC equivocal (2+) cases. This approach is influenced by considering the higher failure rate, longer procedure time, and higher reagent cost of FISH compared with IHC. ERBB2 staining patterns observed by IHC can sometimes deviate from standard definitions; for instance, certain breast cancer subtypes may exhibit moderate to intense but incomplete staining, such as basolateral or lateral patterns, and still test positive for *ERBB2* amplification. Another example includes cases with intense circumferential membrane staining present in 10% or less of tumor cells, which are typically classified as equivocal.^3^ Finally, the 2007 American Society of Clinical Oncology/College of American Pathologists guidelines recommend an initial ERBB2 assessment by IHC using a semiquantitative scoring system and confirmed by FISH in all IHC 2+ cases.^3^

This study aimed to evaluate the utility of the HScore, calculated using ImageJ software, in determining ERBB2 status among patients with breast cancer and IHC 2+ equivocal results. Given that *ERBB2* overexpression or amplification is prevalent in 15% to 20% of invasive breast cancers and associated with aggressive disease, accurate assessment of ERBB2 status is critical for timely and effective treatment. Moreover, the use of anti-HER–targeted agents extends beyond breast malignancies, showing benefit in tumors present in gastric, biliary tract, colorectal, lung, uterine, and other cancers.^13^

We found that the overall mean HScore of our population was 2.65, with the ERBB2-positive cases exhibiting a higher mean HScore of 3.06 compared with 2.62 in ERBB2-negative cases. Although the HScore was generated using ImageJ software to reduce the subjectivity commonly seen with manual evaluation, the process is not entirely objective. For instance, users must still determine the threshold settings within ImageJ, a step that introduces individual judgment into the analysis of the area stained and its intensity. The ROC analysis revealed a moderate AUC of 0.668, indicating that the HScore has limited accuracy as a stand-alone classifier between ERBB2-positive and ERBB2-negative cases, and it cannot replace the gold standard of FISH testing. Moreover, the optimal cutoff identified achieved a sensitivity of 0.77 and a specificity of 0.58. This suboptimal specificity highlights a substantial risk of false positives, which could lead to misclassification and inappropriate treatment decisions.

Unfortunately, data in the literature on using quantitative methods such as the HScore to improve the assessment of ERBB2 status are limited, particularly in IHC 2+ cases. Similar to our study, the MembraneQuant study^14^ used digital image analysis on 15 breast cancer cases with IHC 2+ equivocal results. Their results showed a strong correlation between higher HScore values and *ERBB2* gene amplification, as confirmed by FISH. Although this finding suggests that digital HScore analysis could help predict which cases would test positive on FISH and possibly reduce the need for additional FISH testing, the statistical significance was not quite reached.^14^

Moreover, the moderate AUC observed in our study suggests that the HScore alone is insufficient for reliable classification. Further research is needed to refine this method, aiming to improve automated image analysis to reduce subjectivity and validating this method across multiple centers. Alternatively, researchers could potentially integrate additional diagnostic parameters—novel molecular or imaging biomarkers that may offer improved clinical utility. For now, FISH testing remains essential for confirming ERBB2 status in cases where IHC results are equivocal.

Including ERBB2 IHC 0, 1+, and 3+ cases as internal controls showed that higher HScore values generally correspond to higher ERBB2 protein expression, supporting the image-based scoring method. Immunohistochemistry 3+ cases had the highest mean HScore values, while lower-expression categories had lower values. Despite this trend, there was considerable overlap in HScore distributions among IHC 0, 1+, and 2+ cases. This overlap indicates that staining intensity and extent, as measured by HScore, do not reliably distinguish lower from equivocal expression for individual classification. Although HScore reflects ERBB2 expression at the group level, its accuracy is limited for clinically important equivocal cases, where distinguishing ERBB2-positive from ERBB2-negative status is critical. Therefore, relying solely on HScore could increase the risk of misclassification, underscoring the need for confirmatory testing, such as FISH, to guide accurate ERBB2 assessment and treatment decisions.

### Clinical implications

Identifying ERBB2 status accurately is vital for guiding treatment decisions, particular given the increasing use of targeted therapies for ERBB2-positive cancers, including breast, esophageal, and gastric cancers. The integration of HScore values could help reduce associated costs and expedite the initiation of treatment, but based on our study results, the HScore is not a reliable tool for accurate testing because a single test may result in possible misclassification and the need for confirmatory testing.

### Limitations and future directions

This study has several limitations. Due to its retrospective nature, there is the possibility of inherent bias. Our relatively small sample size of ERBB2-positive cases also weakens the statistical power while limiting the extrapolation to other settings. Furthermore, the data were collected from a single institution, which may restrict generalizability of the results. Future research should focus on larger, multicenter studies to validate our conclusions and explore the integration of the HScore with other biomarkers or imaging techniques to enhance predictive accuracy.

If researchers can address these challenges, the HScore may become a valuable addition to current diagnostic tools for ERBB2-positive malignancies. With the improvements mentioned here, the HScore could help refine treatment strategies and improve patient outcomes. Notably, a new treatment approach has been introduced for patients classified as ERBB2 IHC 1+ or 2+ with negative FISH results, who may now be eligible for FDA-approved ERBB2-low therapies in the metastatic setting^15^; this approach is still in phase 2 clinical trials for earlier stages.^16^ Therefore, the standard dichotomic classification of ERBB2 may eventually become obsolete and a numerical value may be more representative of the potential response to ERBB2-directed therapies.

## Conclusion

This study evaluated the utility of the HScore, calculated using ImageJ software, as a quantitative tool for determining ERBB2 status in breast cancer cases with equivocal IHC results. Our findings revealed that although the HScore demonstrated a moderate correlation with ERBB2 status, as indicated by its ability to differentiate between ERBB2-positive and ERBB2-negative cases to some extent, its predictive accuracy was insufficient for clinical application in equivocal cases of ERBB2 IHC 2+. In particular, the AUC of the ROC curve analysis highlighter the limitations of the HScore in achieving a consistently reliable classification. The suboptimal sensitivity and specificity, along with its moderate discriminatory ability, suggests that relying solely on the HScore might result in misclassification of ERBB2 status, potentially leading to inappropriate therapeutic decisions.

Although the HScore is a promising and cost-effective method, its current constraints emphasize the continued necessity of confirmatory testing in equivocal ERBB2 IHC 2+ results. More research is needed to enhance the current HScore approach.

## Data Availability

The datasets generated or analyzed during the current study are not publicly available to protect patients’ information but are available from the corresponding author on reasonable request.
